# Relationship between depression and psychological well-being among persons with diabetes during COVID-19 pandemic: diabetes distress as a mediator

**DOI:** 10.1007/s40200-022-01025-z

**Published:** 2022-04-06

**Authors:** Wojujutari Kenni Ajele, Oyeyemi Bukola Babalola, Egbe Emmanuel Idehen, Teslim Alabi Oladejo

**Affiliations:** 1 Department of Psychology, Obafemi Awolowo University, Osun State, Ile-Ife, Nigeria; 2 Department of Mental Health, Federal Medical Centre, Lokoja, Nigeria

**Keywords:** Psychological well-being, Depression, And diabetes distress

## Abstract

**Purposes:**

The study examined the relationship between depression, diabetes distress and psychological well-being and also assessed the mediating role of diabetes distress on depression relationship with psychological well-being among persons with diabetes during the covid-19 pandemic.

**Methods:**

The study conducted a cross-sectional survey design. A total of 223 (age 35 to 73 years, mean = 53.26 years and SD = 11.05 years) people living with diabetes who are registered patients and were attending the clinic in Department of medicine, Ondo State Specialist Hospital, Okitipupa were selected for the study using the convenient sampling technique. The data were analysed using Pearson Multiple correlation and mediation model 4 of PROCESS macro. The analyses were carried out with ROCESS macro for IBM/SPSS Version 25.0.

**Results:**

Showed psychological well-being has negative significant relationship between diabetes distress (*r* = −0.42, *p* < .05) and depression (*r* = 0.52, *p* < .05) among persons with diabetes during covid-19 pandemic. The result showed significant and negative direct relationship between depression and diabetes distress (β = −0.47, p < 0.05), 95% Cl: = −0.60 (−0.34). The results also showed significant direct relationship depression and psychological well-being (β = 0.36, p < 0.05), 95% Cl: = 0.26 (0.47) and further that diabetes distress significantly mediate indirect relationship between depression and psychological well-being among persons with diabetes during covid-19 pandemic (β = −0.19, *p* < .05), 95% Cl: = 0.29 (−0.09).

**Conclusion:**

Depression and diabetes distress associated with the psychological well-being of persons with diabetes during covid-19 pandemic and diabetes distress may play vital role on the association between depression and with the psychological well-being of persons with diabetes during covid-19 pandemic.

## Introduction

The devastation of covid-19 infection can obliterate the psychological well-being of persons with diabetes. Covid-19 infected persons diabetes were found to be suffered a strain of coronavirus called Severe Acute Respiratory Syndrome Coronavirus 2 (SARS-CoV-2) [8]. Studies showed that diabetes is a common risk factor of covid-19 and 51% persons with diabetes who suffered covid-19 experienced hyperglycemia [4, 11, 13]. Evidence showed that persons with diabetes experiencing poor psychological well-being [10, 12]. The psychological well-being of persons with diabetes that involves their ability to experience deep happiness, resilience, adequate diabetes-related self-care, good decision-making can be affected by diabetes distress and depression during covid-19 pandemic.

Psychological well-being is about perceived life going well satisfactory by the individual. It is the combination of feeling good and functioning effectively [3]. Prior to the covid-19 pandemic, studies showed that depression significantly decreased the psychological well-being of persons with diabetes [6, 22]. Evidence showed that psychological disorders such as anxiety and depression were found to be common among persons with diabetes, which decreased their psychological well-being [17, 22]. Studies that examined the relationship between depression and psychological well-being were more documented among non-diabetes individual than persons with diabetes [5, 26].

The presence of diabetes distress in depression may worsen the general well-being of persons with diabetes. Diabetes distress involved the emotional burden (fear about managing the demands of diabetes overtime), regimen distress (feeling that they are failing by managing their diabetes properly via feeding regimen, medication regimen, adequate exercising, and self-monitoring of blood glucose levels), interpersonal distress (worries about receiving sufficient support from family and friends) and physician distress (worries about healthcare and receiving expert medical care and support from health care providers) that stunts their psychological resilience to easily manage their present health condition [20].

Severe diabetes distress may be associated with adverse medical and psychological outcomes such as suboptimal self-management (reduced physical activity, poor eating habits, contrivance of medication instructions, and irregular blood glucose checks), elevated Haemoglobin, and frequent and severe hypoglycaemia; all of which may reduce the general well-being of diabetics [2, 18]. Alvani, Zaharim & Kimura, [1] study showed that diabetes distress significantly associated with psychological well-being of persons with diabetes. Peyrot et al., [19] found significant relationship between individual with worst psychological well-being and diabetes- related distress in persons with diabetes. Rahimi, et al., [21] study revealed significant mediating role of resilience and diabetes distress in relationship between depression and treatment adherence. Van Bastelaar, et al. [25] research found diabetes-specific emotional distress as mediating association between depression and glycaemic control.

### Objectives of the study

The main objective was to assess psychological well-being in persons with diabetes during the covid-19 pandemic because it can devastate their psychological well-being. The specific objectives were to examine the relationship between depression, diabetes distress and psychological well-being also assessed the mediating role of diabetes distress on depression relationship with psychological well-being among persons with diabetes during the covid-19 pandemic.

### Design

The study conducted a cross-sectional survey design. Primary data was collected through the administration of a set of standardised psychological scales on a convenient sample of the study population. Also, patients’ data such as diagnosis, and socio-demographic were collected from their medical files. Each file contains patient’s age, sex, and type of diabetes.

### Participants

A total of 223 diabetes patients receiving follow-up clinical treatment in medicine unit, State specialist Hospital, Okitipupa were purposively selected for the study. Ondo State Teaching Hospital, Akure and Federal Medical Centre, Lokoja provide wide range of medical services to patients in different departments. A total of 223 (165 male and 58 female) participants were selected for the study using the convenient sampling technique whereby only patients available on four consecutive clinic days were approached individually and those who consented were included in the study.

The sample comprised of 118 (52.9%) persons with T2DM and 105 (47.1%) and person with T1DM participants in study. The age of the 223 respondents ranged from 35 to 73 years, mean = 53.26 years and SD = 11.05 years.

## Instruments

### Scales of psychological well-being

Scales of Psychological Well-Being (SPWB) is a structured, self-report instrument based on the six dimensions of psychological well-being and developed by Ryff [23, 1995]. The SPWB consists of 18 items phase in the form of sentences with which respondents could agree or disagree. Each item is respondent to on 6-point Like-type scale ranging from ‘strongly disagree’ to ‘strongly agree’. These responses were scored as follows: strongly disagree = 1, moderately disagree = 2, slightly disagree = 3, slightly agree = 4 moderately agree = 5 and strongly agree = 6.

The SPWB could be scored for its six (6) dimensions of the scales are autonomy, positive relationship with other, environment mastery, self-acceptance, personal growth, and purpose in life. The self-acceptance dimension assesses positive attitudes held toward the self” (Akin, 2008). It can also be scored in such a way as to obtain a global psychological well-being.

In this case items scores are summarized across all 18 items to obtain a total. This scoring pattern was adopted in this study. The higher these total scores the better the psychological well-being of the respondent. Internal consistency values provided by Dierendonck, (2005) for the 18 items scale were significantly higher than the values of other versions; reliability coefficient of sub-scales range between 0.72 (personal growth) and 0.81 (self-acceptance, autonomy and purpose in life). The internal consistency reliability coefficients as reported by Ryff [23] ranges from .86 to .93 for the six sub-scales. Reliability coefficient of .63 for the entire scale was found by Onyedibe et al. [15] in Nigeria. The SPWB was used also Oyeleke and Pius [16] on Children with Disabilities in South-Western Nigeria. The current study found reliability coefficient of 0.57 for persons with diabetes.

## Center for Epidemiologic Studies-Depressed Mood Scale (CES-D)

The Center for Epidemiologic Studies Depression Scale (CES-D) was developed by Radloff (1977). The CES-Dis 20-item self-report depression scale was developed to identify depression in the general population. Each item is rated on a 4-point Likert-type scale ranging from zero to three. Estimated administration times range from seven to 12 min (the higher figures being for elderly people) [7]. The CES-D is easily scored by reverse-scoring items 4, 8, 12, and 16, and then summing the scores on all items. This produces a range of zero to 60 with higher scores indicating greater depression.

The CES-D has very good internal consistency with alphas of roughly 0.85 for the general population, and 0.90 for the psychiatric population [14]. Split-half and Spearman-Brown reliability coefficients ranged from 0.77 to 0.92. The CES-D has fair stability with test-retest correlations that range from 0.51 to 0.67 (tested over two to eight weeks) and 0.32 to 0.54 (tested over 3 months to one year) [14].

### Diabetes distress scale (DDS)

Diabetes Distress (DDS) was developed by Polonsky, et al. [20]. The DDS is a 17-item self-report measure designed to assess diabetes distress. The responses to each item are rated on a 6-point Likert-type scale (1 = not a problem, 2 = a slight problem, 3 = a moderate problem, 4 = somewhat serious problem, 5 = a serious problem, and 6 = a very serious problem). The minimum and the maximum scores of the scale were 17 and 102. In this regard, higher scores indicate high distress. Polonsky et al. [20] found high internal consistency of Cronbach’s (α  =  0.87) for the whole DDS. Lin et al. [9] reported good reliability (Cronbach’s α  =  0.81) for DDS. Schmitt et al. [24] reported good reliability Cronbach’s (α  = 0.89) for DDS. The overall DDS-17 Cronbach’s α = 0.92, and the Cronbach’s α values for the subscales ranged from 0.784 to 0.859 as Chin et al. (2017).

### Procedure

Ethical clearance was obtained from the Research Ethics Committee of the hospital. Participants gave informed consent before they were enrolled in the study. The researchers were introduced by the consultant to the nurses, patients and other members of the staff in the department of endocrinology. This was to ensure the maximum support of the nurses and other staff of the hospital. The study questionnaire was administered individually by the researchers on clinic days within the hospital premise. The participants filled questionnaires during their waiting time to see their doctors for consultations. In order to ensure an anonymous process while collecting the data, a drop box was placed in the waiting room. Participants were informed about the drop box and the importance of anonymity. The study data were collected between 8th of April to 22th of July, 2021.

### Data analysis

The data were analysed using Pearson Multiple correlation and mediation model 4 of PROCESS macro. The analyses were carried out with ROCESS macro for IBM/SPSS Version 25.0.

## Results

Table 1 presented the results of study hypothesis that states there is no relationship between diabetes distress, depression and psychological well-being among persons with diabetes during covid-19 pandemic. The results showed psychological well-being negative and significant relationship with diabetes distress (*r* = −0.42, *p* < .05) and depression (*r* = 0.52, *p* < .05). The alternative hypothesis which state there is significant r relationship between diabetes distress depression and psychological well-being among persons with diabetes during covid-19 pandemic was accepted, while the null hypothesis was rejected.

**Table 1:** Summary of multiple Pearson correlation analysis between the diabetes distress depression and psychological well-being (*N =* 223)

Variables	Mean (SD)	1	2	3
1. Diabetes Distress	60.61 (29.51)	1		
2. Depression	36.24 (27.16)	−0.43^**^	1	
3. Psychological Well-Being	32.78 (23.68)	−0.42^**^	0.52^**^	1

Notes

Table 2 presented the results of the hypothesis that states there is no significant mediating role of diabetes distress on the relationship between depression and psychological well-being in persons with diabetes during covid-19 pandemic. This hypothesis was tested with Model 4 mediation, moderation, and conditional process analysis (Hayes, 2018). Diabetes distress among persons with diabetes during covid-19 pandemic was explained by the model at a rate of 19% (R^2^ = 0.19) of the total variance of depression, (F_1, 221_) 51.15, p < 0.05. Also, Psychological well-being among persons with diabetes during covid-19 pandemic was explained by the model at a rate of 19% (R^2^ = 0.19) of the total variance of depression and diabetes, (F_1, 220_) 50.24, *p* < .05.

**Table 2:** Summary of mediation analysis diabetes distress and depression on psychological well-being (model 4 of PROCESS macro; *N =* 223)

Explained Variables						
	Diabetes Distress			Psychological Well-being		
Model	β	*SE*	95% CI	Β	*SE*	95% CI
			LLCI (ULCI)			LLCI (ULCI)
Constant	77.67**	2.98	71.80(83.55)	31.18**	4.45	22.41(39.94)
Depression	−0.47**	0.07	−0.60(−0.34)	0.36**	0.05	0.26 (0.47)
Diabetes Distress				−0.19 **	0.05	0.29(−0.09)
	**R**	0.43		**R**	0.24	
	**R**^**2**^	0.19		**R**^**2**^	0.06	
	***F(df)***	*F*(1, 221) = 51.15 **		***F(df)***	*F*(2, 220) = 50.24 **	

**Fig. 1:**
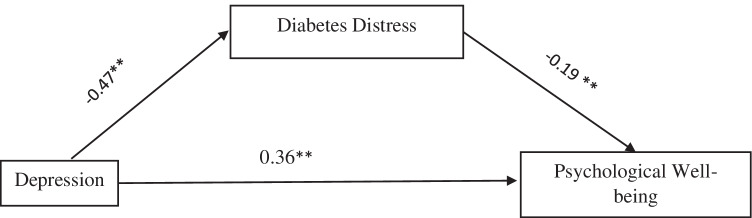
Theoretical research model with standard coefficients (β) Model 4

Mediation analysis presented in Table 2 revealed significant and negative direct relationship between depression and diabetes distress (β = −0.47, *p* < 0.05), 95% Cl: = −0.60(−0.34) see Fig. 1. The results also showed significant direct relationship depression and psychological well-being (β = 0.36, *p* < 0.05), 95% Cl: = 0.26 (0.47) and further revealed significant indirect relationship between depression and psychological well-being which occur via diabetes distress among persons with diabetes during covid-19 pandemic (β = −0.19, *p* < .05), 95% Cl: = 0.29 (−0.09) see Fig. 1.

## 4. Discussion

The present study examined the relationship between depression, diabetes distress and psychological well-being among persons with diabetes during Covid-19 pandemic and also assessed the mediating role of diabetes distress on depression relationship with psychological well-being among persons with diabetes during the covid-19 pandemic in Nigeria.

The study results showed that psychological well-being has negative significant relationship between diabetes distress and depression which is congruent with existing literature of Ramkisson et al. [22] that found strong negative correlation between anxiety and depression and psychologically well-being which pointed in their study that an increase in anxiety and depressive features results in a decrease in psychological well-being. On the other hand, the study results are in accordance with the previous study which established that relationship between emotional distress, psychological well-being and depression of people with diabetes [6].

The study results also supported findings that showed the existing relationship between depression and psychological well-being among non-diabetes persons. Young [26] found significant relationship between depression and psychological well-being mediated by college student’s negative emotional experiences. Joseph and De Guzman [5] found significant relationship between depression and psychological well-being that impaired the mental health of adolescents.

The study results also revealed the diabetes distress significantly mediate indirect relationship between depression and psychological well-being among persons with diabetes during covid-19 pandemic. The study results supported a previous finding by Rahimi et al. [21] who concluded that depression has an indirect effect on the adherence to treatment by mediating role of resilience and diabetes distress. This study results also is in the accordance with study by Van Bastelaar, et al. [25] who admitted diabetes-specific emotional distress as mediating association between depression and glycaemic control.

## Conclusions

Depression and diabetes distress associated with the psychological well-being of persons with diabetes during covid-19 pandemic and diabetes distress may play vital role on the association between depression and with the psychological well-being of persons with diabetes during covid-19 pandemic.
